# Novel insights into the synergistic effects of selenium nanoparticles and metformin treatment of letrozole - induced polycystic ovarian syndrome: targeting PI3K/Akt signalling pathway, redox status and mitochondrial dysfunction in ovarian tissue

**DOI:** 10.1080/13510002.2022.2160569

**Published:** 2023-01-20

**Authors:** Hanem M. Rabah, Darin A. Mohamed, Reham A. Mariah, Sarah R. Abd El-Khalik, Haidy A. Khattab, Norhan A. AbuoHashish, Amal M. Abdelsattar, Mohamed A. Raslan, Eman E. Farghal, Amira K. Eltokhy

**Affiliations:** a Medical Biochemistry Department, Faculty of Medicine, Tanta University, Tanta, Egypt; b Histopathology Department, Faculty of Medicine, Tanta University, Tanta, Egypt; c Medical Physiology Department, Faculty of Medicine, Tanta University, Tanta, Egypt; d Pharmacology Department, Faculty of Medicine, Tanta University, Tanta, Egypt; e Anatomy Department, Faculty of Medicine, Tanta University, Tanta, Egypt; f Gynecology and Obstetrics Department, Faculty of Medicine, Tanta University, Tanta, Egypt; g Clinical and Chemical Pathology Department, Faculty of Medicine, Tanta University, Tanta, Egypt

**Keywords:** Polycystic ovary syndrome, obesity, insulin resistance, SeNPs, PI3K/Akt pathway, metformin, mitochondrial dysfunction, oxidative stress

## Abstract

**Purpose:**

Polycystic ovary syndrome (PCOS) has a series of reproductive and metabolic consequences. Although the link between PCOS, IR, and obesity, their impact on the pathogenesis of PCOS has yet to be determined. Dysfunction of PI3K/AKT pathway has been reported as the main cause of IR in PCOS. This study purposed to explore the effects of selenium nanoparticles (SeNPs) alone and combined with metformin (MET) in a PCOS-IR rat model.

**Methods:**

After 3 weeks of treatment with SeNPs and/or MET, biochemical analysis of glycemic & lipid profiles, and serum reproductive hormones was performed. Inflammatory, oxidative stress, and mitochondrial dysfunction markers were determined colormetrically. The expression of PI3K and Akt genes were evaluated by Real-time PCR. Histopathological examination and Immunohistochemical analysis of Ki-67 expression were performed.

**Results:**

The results showed that treatment with SeNPs and/or MET significantly attenuated insulin sensitivity, lipid profile, sex hormones levels, inflammatory, oxidative stress and mitochondrial functions markers. Additionally, PI3K and Akt genes expression were significantly upregulated with improved ovarian histopathological changes.

**Conclusion:**

Combined SeNPs and MET therapy could be potential therapeutic agent for PCOS-IR model via modulation of the PI3K/Akt pathway, enhancing anti-inflammatory and anti-oxidant properties and altered mitochondrial functions.

**Highlights**
- The strong relationship between obesity, insulin resistance, and polycystic ovarian syndrome.
- Disturbance of the PI3K/Akt signaling pathway is involved in the progression of polycystic ovary syndrome-insulin resistance (PCOS-IR).
- In PCOS-IR rats, combined SeNPs and metformin therapy considerably alleviated IR by acting on the PI3K/Akt signaling pathway.
- The combination of SeNPs and metformin clearly repaired ovarian polycystic pathogenesis and improved hormonal imbalance in PCOS-IR rats.

## 1. Introduction

Polycystic ovarian syndrome (PCOS) is a prevalent endocrine disorder that affects 9-18% of women of fertile age. It is a multifaceted endocrine and metabolic disorder associated with oligo/anovulation, clinical or biochemical hyperandrogenism, and polycystic ovarian changes [1]. In addition, women with PCOS have a higher prevalence of other comorbidities such as obesity, dyslipidemia, hypertension, metabolic syndrome, and type 2 diabetes mellitus, raising the risk of cardiovascular disease [2].

Both PCOS and obesity have alarmingly high prevalence rates, with a worldwide prevalence of 35% in females. Furthermore, several studies have identified insulin resistance (IR) as the fundamental link attributed to these conditions [3]. Therefore, it is crucial to understand the complex pathophysiologic cross-talk between PCOS, IR, and obesity. Indeed, IR and hyperinsulinemia play a critical role in the underlying molecular mechanisms involved in androgenic hypersecretion. Conversely, hyperandrogenemia may exacerbate IR and hyperinsulinemia resulting in a vicious cycle of pathological scenarios that leads to PCOS-IR [4].

The insulin signalling pathway is controlled by an intricate, highly regulated system. The phosphatidylinositol 3-kinase/protein kinase B (PI3K/Akt) pathway plays a pivotal role in regulating insulin's impacts on metabolism [5]. When insulin binds to the insulin receptor, the insulin receptor substrate (IRS) recruits and activates PI3K to generate the second messenger lipid PI-3,4,5-triphosphate (PIP3), which enhances the recruitment and activation of Akt. After that, Akt triggers downstream pathways that regulate cell survival, glucose, and lipid metabolism [6]. The PI3K/Akt signalling pathway has been widely reported to have a primary effect on ovarian follicular granulosa cells. Thus, impairment of the PI3K/Akt signalling pathway is implicated in the pathophysiology of IR and PCOS [7].

Mitochondria are essential organelles for intracellular redox status as they generate ROS and release other intermediates that are commonly neutralized by the anti-oxidant system. There is clear evidence of mitochondrial dysfunction in PCOS patients, as mitochondrial structure, dynamics, biogenesis, and mitochondrial membrane potential (MMP) are disrupted [8]. Previous research has revealed that PCOS development and progression are influenced by mitochondrial dysfunction. Mitochondrial mutations, dysregulated mitophagy, decreased ATP production and released ROS all play a role in other related symptoms, particularly IR and obesity [9].

Aromatase is a rate-controlling enzyme that is required for the conversion of androgens into estrogens. Letrozole, on the other hand, is an effective aromatase inhibitor. It prevents testosterone from being converted to estrogen in the ovary, resulting in hyperandrogenism and polycystic ovarian alterations, and has been frequently utilized as a PCOS rat model [10]. Moreover, Obesity and IR have been linked to a high-fat diet, which has been shown to trigger a hypothalamic inflammatory response and contribute to improper glucose and lipid metabolism [11]. Therefore, in our study, letrozole was paired with a high-fat diet to establish a PCOS-IR rat model.

The initial line of treatment for PCOS-IR was metformin hydrochloride. It can potentially improve IR and induce ovulation [12]. However, it frequently causes gastrointestinal side effects such as anorexia, nausea, diarrhoea, flatulence, metallic taste, and abdominal pain that would diminish the patient's compliance to treatment, and the long-term outcome would be inadequate [13].

Because of their multifunctional biological activities, several nanoparticles (NPs) are now used as an alternative therapy for various diseases [14]. Selenium (Se) is an important anti-oxidant and anti-inflammatory micronutrient that can reduce reactive oxygen species (ROS) production by increasing the activity of selenoproteins [15]. Additionally, selenium nanoparticles (SeNPs) have become one of the most prominent metal oxide nanoparticles in biological applications because of their outstanding biocompatibility, low toxicity, and low cost [16]. SeNPs have shown promise in biomedicine, exhibiting excellent biomedical applications such as anticancer, drug delivery, antimicrobial, anti-oxidant, anti-diabetic, and nutritional supplements [17]. Previous studies proved that treating diabetic rats with SeNPs improved the metabolic state and reduced IR [18].

In the current study, we aimed to investigate the pathophysiology and metabolic dysfunction of PCOS in a PCOS-IR rat model and to evaluate the effect of SeNPs treatment alone or combined with metformin as a promising treatment that helps to overcome IR and most PCOS symptoms.

## 2. Materials and methods

### 2.1. Drugs and chemicals

Letrozole was obtained from Natco Pharma Limited, Hyderabad, India, metformin (MET) and carboxymethyl cellulose (CMC) were supplied by Sigma Pharmaceutical Company, Egypt.

SeNPs, with 80 nm particle size and CAS #:7782-49-2, were obtained from Sigma Aldrich (St., Louis, MO, USA). The prepared SeNPs, according to the manufacturer, were characterized to ensure consistent materials.

SeNPs were sonicated for 10 min in a sonicator for better dispersion before administration. All other reagents and chemicals used were of analytical grade obtained from Biodiagnostic Co. (Cairo, Egypt).

### 2.2. Experimental animals

A total of 75 virgin adult female albino rats aged 4–6 weeks weighing 80–120 grams were obtained from the Animal House of the Faculty of Medicine, Tanta University. Before the study, rats were given three days to adjust to their new environment. They were kept under constant environmental conditions of temperature (23 ± 2°C) and a 12 h light/dark cycle along the experimental period. This study was done in agreement with the guiding ethics of the Ethical Committee of Medical Research, Faculty of Medicine, Tanta University, adopting the National Institutes of Health guide for the care and use of Laboratory animals (NIH Publications No. 85-23, revised in 1996).

### 2.3. Experimental design

Rats were randomly divided into two groups:

**Control group (n = 15):** rats were given a control normal diet (fat 5%, carbohydrates 65%, proteins 20.3%, fibre 5%, salt mixture 3.7% and vitamin mixture 1%).

**High fat diet group (n = 60):** rats were fed a high-fat diet (HFD) containing (fat 46%, carbohydrates 24%, proteins 20.3%, fibre 5%, salt mixture 3.7%, and vitamin mixture 1%) for six weeks. HFD constituents were purchased from El-Gomhoria Company, Cairo, Egypt and were preserved at 4°C until used. Dietary composition (g/kg diet) was according to the formula of Kim et al. with some modifications [19]. Rats’ body weight and naso-anal length were measured weekly, as well as Lee’s index (cube root of body weight (g)/naso-anal length (cm)) [20].

For induction of a PCOS-IR model, letrozole 1 mg/kg/day dissolved in 0.5% CMC solution was given by gavage on day 22 of the HFD for the next 21 consecutive days. The control group animals were given the same volume of 0.5% CMC using oral gavage. Vaginal Smears were collected and evaluated microscopically to confirm the induction of PCOS. On day 42 of the HFD and after overnight fasting, blood samples were obtained from the tails of PCOS rats and fasting blood glucose and fasting insulin was measured. Homeostasis model evaluation of insulin resistance (HOMA-IR) was calculated. PCOS-IR model rats were chosen with HOMA-IR > 2.8.

- PCOS-IR rats were designated into four groups, each of 12 rats:

- **PCOS-IR** model group.
- **SeNPs-treated group** (0.5 mg/kg body weight/day for three weeks) [21].
- **MET-treated group** (300 mg/kg/day for three weeks).
- **Combined SeNPs + MET-treated group** (50 mg/kg + 300 mg/kg/day for three weeks).

### 2.4. Collection of samples

**Blood Sampling:**

At the end of the prescribed period, all rats were fasting overnight and sacrificed by decapitation under light ether anaesthesia. Blood was taken from retro-orbital plexuses, allowed to clot at room temperature, and then centrifuged for 10 min at 5000 rpm. Sera were separated and divided into small aliquots and frozen at −20°C for further biochemical assay.

**Tissue sampling:**

After decapitation, abdomens were opened; ovarian tissues were divided into two sides, one side immediately perfused in situ with ice-cold saline and stored at −80°C for further analysis of biochemical and mitochondrial parameters. The second side was removed and fixed in formalin before being dried and embedded in paraffin for histopathological examination.

### 2.5. Biochemical analysis

#### 2.5.1. Assay of fasting serum glucose and insulin level

Fasting serum glucose (FSG) level was measured by oxidase method using assay kit (Biodiagnostic, Egypt), and fasting serum insulin level was measured by ELISA technique using rat insulin ELISA kit (Crystal Chem, IL USA, Cat# 90010**)** following the manufacturer's instructions. The HOMA-IR index was calculated according to Bonora et al. [22] as follows:

HOMA-IR index = fasting serum glucose (mg/dL) × 

fasting serum insulin (μIU/L) /405

#### 2.5.2. Assay of lipid profile

Lipid profile, including serum concentrations of total cholesterol (TC), triglycerides (TG) and high-density lipoprotein cholesterol (HDL-C), were measured by colourimetric methods using assay kits (Spinreact, Spain). Low-density lipoprotein cholesterol (LDL-C) concentration was calculated according to Friedewald et al. [23] as LDL-C = TC − HDL-C − (TG/5).

#### 2.5.3. Assay of hormonal profile

The serum levels of estradiol (E2), luteinizing hormone (LH), follicle-stimulating hormone (FSH), and testosterone (T) were determined using ELISA kits: Rat Estradiol ELISA Kit (My BioSource, USA, Cat# MBS843353), Rat LH ELISA Kit (CUSABIO Biotech Co., Ltd., China, Cat# CSB-E12654r), Rat FSH ELISA Kit (CUSABIO Biotech Co., Ltd., China, Cat# CSB-E06869r), and Mouse/Rat Testosterone ELISA Kit (Sigma Aldrich, USA, Cat# SE120089) respectively according to the protocol provided with each kit. In addition, the LH/FSH ratio was measured.

#### 2.5.4. Assay of inflammatory and redox status parameters

The tumour necrosis factor (TNF-α) level was determined using a Rat TNF-α ELISA kit (MyBioSource, USA, Cat# MBS355371) following the manufacturer's protocol.

Ovarian tissue samples were homogenized in ice-cold phosphate buffer (pH 7.4) before being centrifuged for 30 min at 4 °C. The supernatant was used to examine malondialdehyde level (MDA), superoxide dismutase activity (SOD), and reduced glutathione (GSH) level using a commercial kit supplied by Bio Diagnostic, Egypt.

#### 2.5.5. Mitochondrial samples analysis

Half of the ovarian tissues were homogenized in a mitochondrial buffer and then centrifuged for 10 min at 2,000 g. Mitochondria were obtained by centrifuging the supernatants for 20 min at 5,000 g. The obtained mitochondrial fraction used for estimation of; mitochondrial transmembrane potential (MMP or ΔΨm) by the method of Maity et al. [24], electron transport chain (ETC) complex-I activity (Cat# No. ab109721) and mitochondrial ATP levels using a colourimetric ATP assay kit (Cat. No. K354–100, BioVision, Mountain View, CA, USA).

#### 2.5.6. Quantitative measurement of PI3K and Akt genes expression in ovarian tissues

- Total cellular RNA was extracted from ovarian tissue samples using Trizol Reagent (InvitrogenInc., USA). The purity and concentration of extracted RNA were determined using the NanoDrop Spectrophotometer (Implen, USA).
- cDNA was synthesized using RevertAid H Minus Reverse Transcriptase kit (Thermo Scientific, Fermentas, #EP0451) according to the manufacturer's instructions. The isolated cDNA was used to perform qRT-PCR using 2X Maxima SYBR Green/ROX qPCR Master Mix (Thermo Scientific, USA, # K0221) and gene-specific primers (Table 1) using a StepOnePlus real-time thermal cycler (Applied Biosystems, Life Technology, USA). The mRNA levels were normalized to *GAPDH* internal control gene. Relative mRNA expression levels were calculated using the 2^−ΔΔCt^ method [25].

**Table 1.** Forward and reverse primers sequence for candidate genes.

Gene	Primer Sequence
*Akt*	F 5'- TGTCTCGTGAGCGCGTGTTT −3’ R 5'- CCGTTATCTTGATGATGTGCCCGTC −3'
*PI3K*	F 5'- CTTGCCTCCATTCACCACCTCT −3’ R 5'- GCCTCTAATCTTCTCCCTCTCCTTC −3'
*GAPDH*	F 5’- TCTCTGCTCCTCCCTGTTC −3’ R 5'- ACACCGACCTTCACCATCT −3'

### 2.6. Histopathological examination

Ovarian tissues were dissected, fixed for 24 h in 4% paraformaldehyde, and then embedded in paraffin. For histopathological examination, 4 µm thick tissue sections were stained with hematoxylin and eosin (H&E). A light microscope (Olympus, Tokyo, Japan) was used to examine the ovary histopathological lesions

### 2.7. Immunohistochemical analysis of ki-67 antigen expression in ovarian tissues

For antigenic retrieval, the tissue sections were deparaffinized, rehydrated, and pre-treated with 10 mM citrate buffer (pH 6.0). Sections were incubated overnight at 4 °C with anti-Ki67 rabbit monoclonal antibody (Dako, Denmark; dilution 1:50). The tissue sections were incubated with biotinylated secondary antibody, and product visualization was performed with diaminobenzidine substrate. Then the slides were counter stained with Mayer's hematoxylin then, dehydrated, and mounted. The slides were examined by a light microscope; brownish discolouration of the cells indicates a positive reaction. The immunolabeled dark brown areas were counted, and the percentage of the positively stained area was calculated in the granulosa cell layer, theca cell layers, and the interstitial stromal cell layer.

### 2.8. Statistical analysis

Statistical analysis of results, including the mean ± SD, and *p* values, were performed using Statistical Package for Social Science (SPSS) version 23.0 for windows. All data are given as means ± SD. Data were analyzed statistically using one-way analysis of variance (ANOVA) followed by Tukey's post-hoc test for comparison between individual groups. Differences were considered significant at *P* < 0.01.

## 3. Results

### 3.1. Body weight and Lee’s index

As shown in Table 2, the control group encountered a reasonable increase in body weight during the study period. However, variations in weight gain were observed between the untreated PCOS-IR model group and the treated groups. The final body weight of the model group was significantly increased. On the other side, the body weight of SeNPs- and/or MET-treated groups was significantly decreased compared to the untreated PCOS-IR group.

**Table 2.** Body weight, Lee's index, fasting serum glucose, insulin levels and HOMA-IR.

Parameters	Control = (n = 15)	PCOS-IR (n = 12)	MET (n = 12)	SeNPs (n = 12)	SeNPs + MET (n = 12)
Final body weight (g)	168 ± 7.51	370 ± 5.26^a^^,^^c^^,^^d^^,^^e^	287.63 ± 11.7^a^^,^^b^^,^^e^	282.63 ± 8.42^a^^,^^b^	203.92 ± 8.12^a^^,^^b^^,^^c^
Lee's index (g/cm)	305.12 ± 2.61	324.62 ± 2.69^a^^,^^c^^,^^d^^,^^e^	316.28 ± 3.11^a^^,^^b^	314.16 ± 2.53^a^^,^^b^	311.73 ± 1.82^a^^,^^b^
FSG (mg/dl)	87.32 ± 3.81	212.4 ± 9.51^a^^,^^c^^,^^d^^,^^e^	118.61 ± 2.73^a^^,^^b^^,^^e^	120.72 ± 6.16^a^^,^^b^	94.5 ± 5.42^b^^,^^c^
Fasting insulin (μIU/L)	10.64 ± 1.82	32.96 ± 4.15^a^^,^^c^^,^^d^^,^^e^	13.31 ± 2.66^a^^,^^b^^,^^e^	15.26 ± 2.58^a^^,^^b^	11.79 ± 1.37^b^^,^^c^
HOMA-IR	2.29 ± 1.34	17.28 ± 2.14^a^^,^^c^^,^^d^^,^^e^	3.89 ± 1.54^a^^,^^b^^,^^e^	2.97 ± 1.73^b^	2.75 ± 2.53^b^^,^^c^

Data were expressed as mean ± SD, ^a^Significant change vs. control, ^b^Significant change vs. PCOS-IR, ^c^Significant change vs MET, ^d^Significant change vs. SeNPs, ^e^Significant change vs. SeNPs + MET. PCOS, Polycystic ovarian syndrome; MET, Metformin; SeNPs, Selenium nanoparticles. IR, insulin Resistance; FSG, Fasting serum glucose; HOMA-IR, HOMA insulin resistance index.

Compared to the control group, Lee’s index of the PCOS-IR group was significantly increased. Conversely, relative to the untreated group, Lee’s indices of SeNPs- and/or MET-treated groups were significantly decreased.

### 3.2. Fasting serum glucose and insulin levels

As shown in Table 2, there was a significant increase in FSG and insulin levels in the PCOS-IR group compared to the control group; however, all treated groups had a significant reduction. Compared to untreated rats, combined treatment demonstrated more efficient anti-hyperglycemic activity than SeNPs or MET alone, with maximum decrease.

Concerning HOMA-IR, the PCOS-IR group showed significant insulin resistance compared to the control group. Conversely, SeNPs-treated groups had a significant reduction. The combination of SeNPs with MET displayed a more significant anti-IR effect that significantly decreased HOMA-IR compared to untreated rats.

### 3.3. Lipid profile

The untreated PCOS-IR group showed a significant increase in serum TG, TC, and LDL-C levels and a decrease in serum HDL-C levels compared to the control group. However, SeNPs- and/or MET-treated groups displayed a significant decline in serum TG, TC, and LDL-C with a marked increase in HDL-C level compared to the untreated group, as demonstrated in Table 3.

**Table 3.** Serum lipid profile among studied groups.

Parameters	Control (n = 15)	PCOS-IR (n = 12)	MET (n = 12)	SeNPs (n = 12)	SeNPs + MET (n = 12)
TG (mg/dL)	61.68 ± 6.18	107.37 ± 4.47^a^^,^^c^^,^^d^^,^^e^	87.12 ± 2.92^a^^,^^b^^,^^e^	83.72 ± 6.81^a^^,^^b^	64.11 ± 3.82^b^^,^^c^
TC (mg/dL)	83.55 ± 5.21	134.89 ± 6.43^a^^,^^c^^,^^d^^,^^e^	98.52 ± 2.67^a^^,^^b^	102.64 ± 4.22^a^^,^^b^	96.45 ± 3.51^a^^,^^b^
HDL-C (mg/dL)	52.61 ± 6.37	23.14 ± 3.45^a^^,^^c^^,^^d^^,^^e^	42.53 ± 5.16^a^^,^^b^	39.33 ± 5.71^a^^,^^b^	48.61 ± 3.08^a^^,^^b^
LDL-C (mg/dL)	22.31 ± 4.52	90.43 ± 2.54^a^^,^^c^^,^^d^^,^^e^	39.23 ± 2.85^a^^,^^b^	47.37 ± 4.24^a^^,^^b^	35.25 ± 2.79^a^^,^^b^

Data were expressed as mean ± SD, ^a^Significant change vs. control, ^b^Significant change vs. PCOS-IR, ^c^Significant change vs. MET, ^d^Significant change vs. SeNPs, ^e^Significant change vs SeNPs + MET. PCOS, Polycystic ovarian syndrome; MET, Metformin; SeNPs, Selenium nanoparticles. IR, insulin Resistance; TG, triacylglycerol; TC, total cholesterol; HDL-C, high-density lipoprotein cholesterol; LDL-C, low-density lipoprotein cholesterol.

### 3.4. Serum levels of reproductive hormones

As shown in Table 4, the PCOS-IR model group revealed significantly decreased estradiol and increased testosterone levels compared to the control group. Although treatment with MET improved such impairment, there was already a significant difference between the MET-treated group and the control group. However, SeNPs treatment significantly prohibited these disturbing levels compared to untreated rats. On the other side, there was no significant difference between SeNPs + MET-treated group and the control group.

**Table 4.** Serum reproductive hormones among studied groups.

Parameters	Control (n = 15)	PCOS-IR (n = 12)	MET (n = 12)	SeNPs (n = 12)	SeNPs + MET (n = 12)
Estradiol (pg/ml)	14.42 ± 1.85	7.51 ± 2.69^a^^,^^c^^,^^d^^,^^e^	10.32 ± 2.21^a^^,^^b^^,^^e^	10.83 ± 2.27^a^^,^^b^^, e^	13.64 ± 1.52^b^^,^^c^^,^^d^
Testosterone (ng/ml)	0.39 ± 2.52	2.87 ± 1.47^a^^,^^c^^,^^d^^,^^e^	0.81 ± 2.33^a^^,^^b^^,^^e^	0.86 ± 1.78^a^^,^^b^^,^^e^	0.42 ± 2.63^b^^,^^c^^,^^d^
LH (mIU/mL)	0.94 ± 0.26	8.72 ± 1.68^a^^,^^c^^,^^d^^,^^e^	3.46 ± 2.48^a^^,^^b^	3.11 ± 2.57^a^^,^^b^	2.11 ± 1.94^a^^,^^b^
FSH (mIU/mL)	4.98 ± 1.25	1.64 ± 1.80^a^^,^^c^^,^^d^^,^^e^	2.30 ± 1.67^a^^,^^b^	2.66 ± 1.18^a^^,^^b^	3.9 ± 1.02^b^^,^^c^
LH/FSH ratio	0.19 ± 0.32	5.31 ± 0.63^a^^,^^c^^,^^d^^,^^e^	1.5 ± 1.84^a^^,^^b^	1.16 ± 0.87^a^^,^^b^	0.54 ± 1.36^b^

Data were expressed as mean ± SD, ^a^Significant change vs. control, ^b^Significant change vs. PCOS-IR, ^c^Significant change vs. MET, ^d^Significant change vs. SeNPs, ^e^Significant change vs SeNPs + MET. PCOS, Polycystic ovarian syndrome; MET, Metformin; SeNPs, Selenium nanoparticles; LH, luteinizing hormone; FSH, follicle stimulating hormone.

In addition, LH and LH/FSH ratio levels in the PCOS-IR group were significantly higher, while FSH levels were significantly lower compared to the control group. On the other hand, LH and LH/FSH ratios were significantly lower in SeNPs and/or MET-treated rats compared to untreated rats, while FSH levels were significantly higher.

### 3.5. Inflammatory and redox status

**Figure 1.**
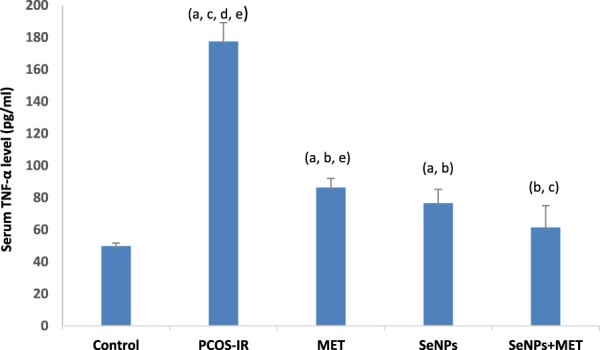
Serum tumour necrosis alpha (TNF-α) level: Data were expressed as mean ± SD, ^a^Significant change vs. control, ^b^Significant change vs. PCOS-IR, ^c^Significant change vs. MET, ^d^Significant change vs. SeNPs, ^e^Significant change vs. SeNPs + MET. PCOS, Polycystic ovarian syndrome; MET, Metformin; SeNPs, Selenium nanoparticles.

As shown in Figure 1, the serum TNF-α level in the PCOS-IR group was significantly higher than in the control group. However, the inflammatory effect was attenuated by SeNPs or MET treatment compared to untreated rats. Furthermore, the anti-inflammatory effect of combined treatment was more potent in terms of serum TNF-α reduction compared to the untreated group.

**Figure 2.**
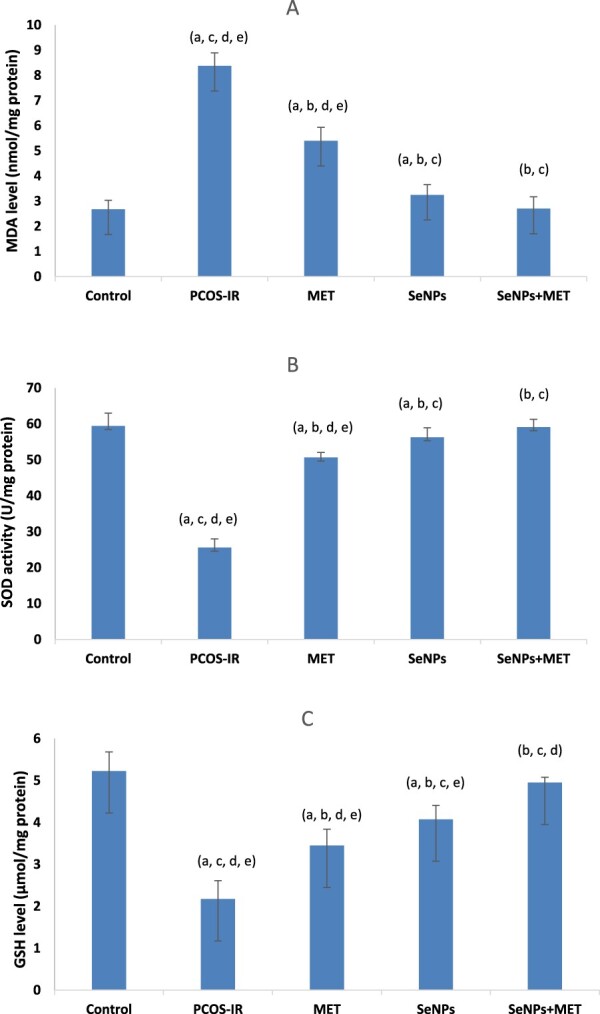
Oxidative stress markers in ovarian tissues: (A) The MDA level (nmol/mg protein). (B) SOD activity (U/mg protein). (C) GSH level (μmol/g protein). Data were expressed as mean ± SD, ^a^Significant change vs. control, ^b^Significant change vs. PCOS-IR, ^c^Significant change vs. MET, ^d^Significant change vs. SeNPs, ^e^Significant change vs. SeNPs + MET. PCOS, Polycystic ovarian syndrome; MET, Metformin; SeNPs, Selenium nanoparticles.

Compared to the control group, the untreated PCOS-IR group experienced significant oxidative stress, as evidenced by an elevation in MDA level, a reduction in GSH level and SOD enzyme activity in the ovarian tissues. On the other hand, treated groups showed improvement in oxidative stress markers, as evidenced by a significant decrease in MDA levels and a significant increase in GSH levels and SOD enzyme activity compared to untreated rats. Combined treatment improved oxidative stress more effectively compared to untreated groups (Figure 2).

### 3.6. Mitochondrial function

Our study showed a significant decline in mitochondrial transmembrane potential (MMP), ATP level, and complex-I activity in the PCO-IR group compared to the control group. On the other side, compared with the PCOS-IR group, the treatment with SeNPs or MET has shown significant correction in the MMP, ATP level, and complex-I activity. However, a marked correction has been observed in SeNPs + MET group as compared with MET or SeNPs single treatment (Table 5).

**Table 5.** Mitochondrial function parameters among studied groups.

Parameters	Control (n = 15)	PCOS-IR (n = 12)	MET (n = 12)	SeNPs (n = 12)	SeNPs + MET (n = 12)
complex I (nmol/min/mg protein)	52.06 ± 3	21.96 ± 2.5^a^^,^^c^^,^^d^^,^^e^	40.66 ± 2^a^^,^^d^^,^^e^	47.73 ± 3.2^b^^,^^c^	49.1 ± 2.1^b^^,^^c^
Mitochondrial ATP (nmol/mg protein)	243.13 ± 7	81.06 ± 15.6^a^^,^^c^^,^^d^^,^^e^	141.4 ± 9^a^^,^^b^^,^^d^^,^^e^	189.8 ± 11.8^a^^,^^b^^,^^c^	232.63 ± 8.8^b^^,^^c^
Mitochondrial transmembrane potential (Fluorescent Unit)	8.16 ± 0.2	2.29 ± 0.31^a^^,^^c^^,^^d^^,^^e^	3.6 ± 0.7^a^^,^^b^^,^^d^^,^^e^	5 ±0.1^a^^,^^b^^,^^c^^,^^e^	6.13 ± 0.3^b^^,^^c^^,^^d^

Data were expressed as mean ± SD, ^a^Significant change vs. control, ^b^Significant change vs. PCOS-IR, ^c^Significant change vs. MET, ^d^Significant change vs. SeNPs, ^e^Significant change vs. SeNPs + MET. PCOS, Polycystic ovarian syndrome; MET, Metformin; SeNPs, Selenium nanoparticles.

### 3.7. Expression of PI3K and Akt genes in ovarian tissues

**Figure 3.**
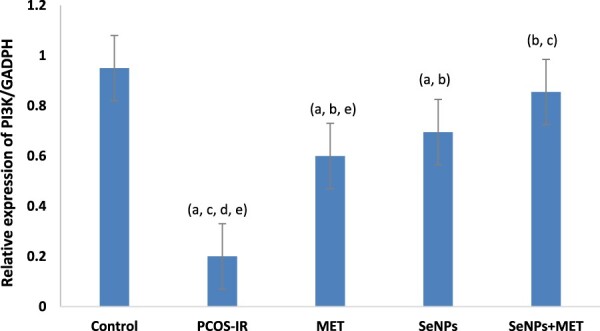
Quantitative Real-time PCR analysis of mRNA expression levels of *PI3K* gene in ovarian tissue among the studied groups. Data were expressed as mean ± SD, ^a^ significant change vs. control, ^b^ Significant change vs. PCOS-IR, ^c^ Significant change vs. MET, ^d^ Significant change vs. SeNPs, ^e^ Significant change vs. SeNPs + MET. PCOS, Polycystic ovarian syndrome; MET, Metformin; SeNPs, Selenium nanoparticles.

**Figure 4.**
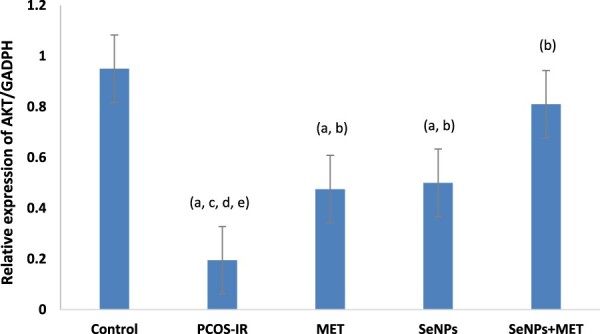
Quantitative Real-time PCR analysis of mRNA expression levels of *Akt* gene in ovarian tissue among the studied groups. Data were expressed as mean ± SD, ^a^ significant change vs. control, ^b^ Significant change vs. PCOS-IR, ^c^ Significant change vs. MET, ^d^ Significant change vs. SeNPs, ^e^ Significant change vs. SeNPs + MET. PCOS, Polycystic ovarian syndrome; MET, Metformin; SeNPs, Selenium nanoparticles.

As illustrated in Figures 3 & 4, the PCOS-IR group showed a significant decrease in the expression of PI3K and Akt genes in the whole ovarian tissue compared to the control group. However, suppression of the insulin signalling pathway after treatment with SeNPs and MET was ameliorated by a significant increase in gene expression compared to untreated rats. Surprisingly, rats treated with a combination of SeNPs and MET had significantly higher gene expression levels in the ovarian tissue than the untreated group.

### 3.8. Histopathological examination

**Figure 5.**
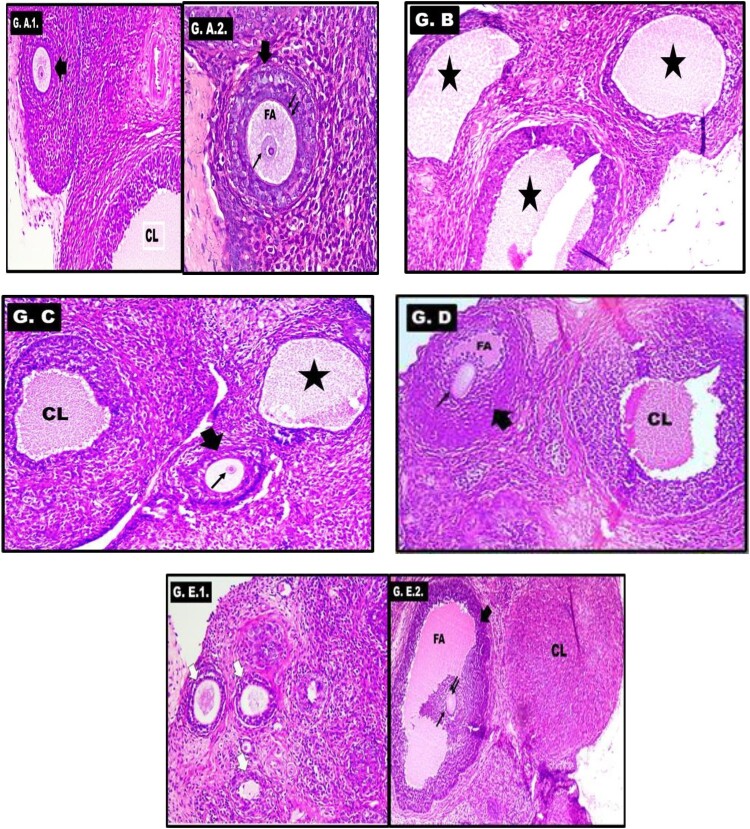
**Histopathological examination of the ovary in different groups: A)** Control group showing the antral follicle (thick arrow) and corpus luteum (CL) (Figure A1). The follicle shows healthy granulosa cells, zona pellucida (double arrow) and oocyte (thin arrow) within the follicular antrum (FA) (Figure A2). **B)** PCOS-IR model group showing multiple follicular cysts (stars) with absent corpus luteum. **C)** MET-treated group showing the antral follicle (thick arrow) with oocyte (thin arrow) and its follicular antrum. The corpus luteum (CL) reappears but with irregular luteal cells compared to the control. A cystic follicle (star) is also seen. **D)** SeNPs-treated group showing antral follicle (thick arrow) with oocyte (thin arrow) but with scanty follicular antrum (FA). The corpus luteum (CL) reappears but with irregular luteal cells. **E)** SeNPs + MET-treated group showing multiple primary follicles (white arrows) (Figure A1). The antral follicle (thick arrow) with oocyte (thin arrow), zona pellucida (double arrows) and its follicular antrum (FA). The corpus luteum (CL) is also seen (Figure A2). All slides are H&E stained. Magnification: × 200.

The ovarian tissue in the control group showed normal ovarian follicles and granulosa cells (Figure 5A). However, the untreated PCOS-IR group showed numerous subcapsular follicular cysts with very thin granulosa cells and no oocytes (Figure 5B). On the other hand, treatment with SeNPs or MET showed lesser cysts with thick granulosa cells and normal follicles (Figure 5C& D). Additionally, in the SeNPs + MET-treated group, there was appearance of oocytes (Figure 5E).

### 3.9. Immuno-histochemical expression of ki-67

**Figure 6.**
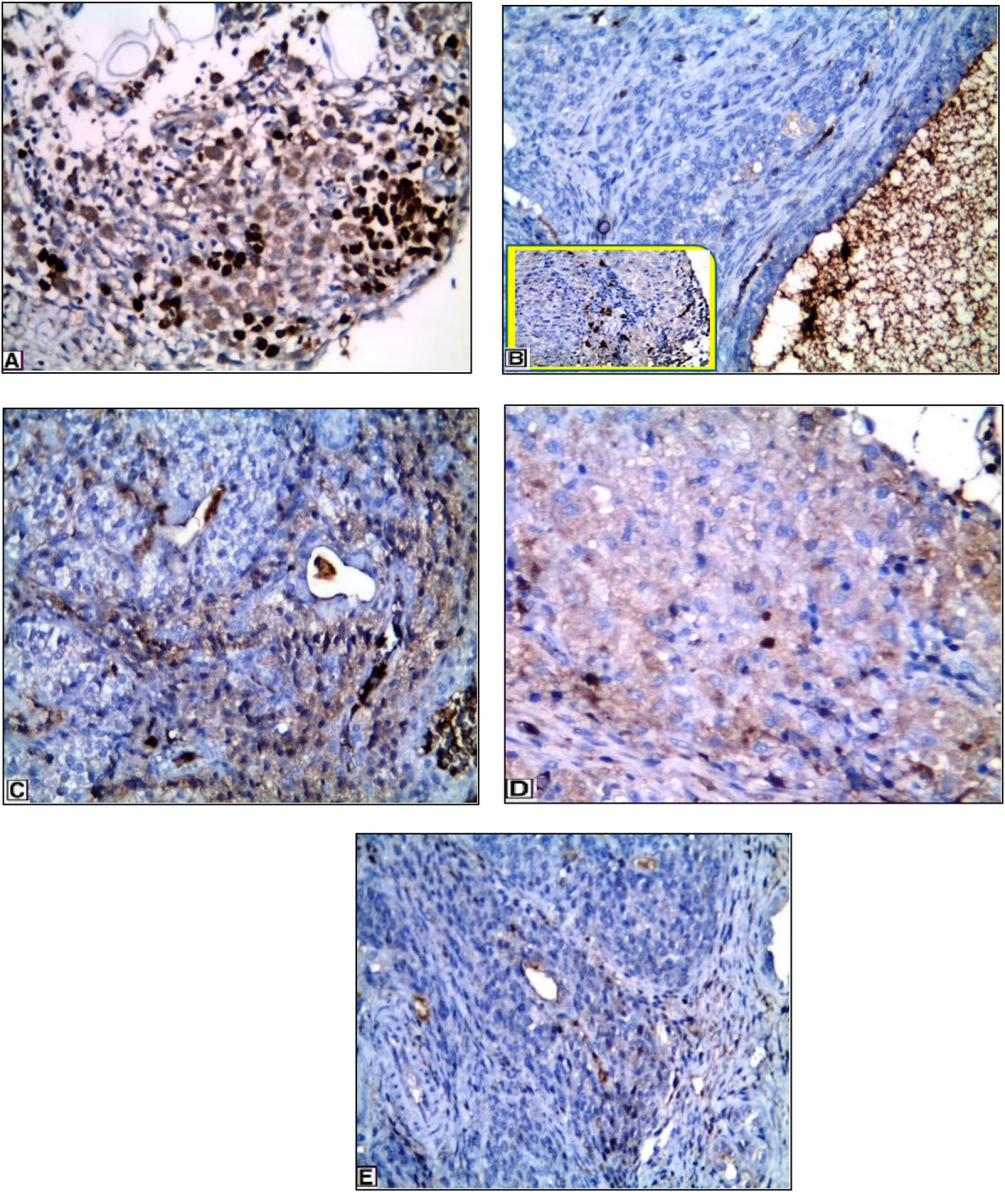
**Immunohistochemical expression of ki-67 in different groups:** A) Control group showing strong immunopositive staining in granulosa cells. B) PCOS-IR model group showing Ki**-**67 nuclear expression mainly in the theca cells and nearly absent in granulosa cells (higher power figure is inserted in the left box). C) MET-treated group showing ki**-**67 expression mainly in granulosa cells. D) SeNPs -treated group showing ki**-**67 expression mainly in granulosa cells. E) SeNPs + MET-treated group showing higher ki**-**67 expression and is present mainly in granulosa cells. Magnification: × 400.

Figure 6 presents the results of the immunohistochemical analysis of Ki-67 and its expression in granulosa cells of the ovaries in various groups. The control group showed high Ki-67 nuclear expression in the granulosa cell layer of the follicles (Figure 6A). The untreated PCOS-IR group showed few Ki-67 nuclear expression mainly in the theca cells and was nearly absent in granulosa cells (Figure 6B). However, SeNPs- and MET-treated groups showed higher Ki-67 nuclear expression and were present mainly in the granulosa cell layer of the follicles (Figure 6C, D &E).

## 4. Discussion

PCOS is a major endocrine disorder of the female reproductive system that has a deleterious effect on women's health-related quality of life [26]. Because of its complexity, PCOS does not have a consistent treatment. However, lifestyle changes, hormonal contraceptives, and some other drugs have been shown to alleviate PCOS symptoms [27]. The purpose of this study was whether SeNPs successfully treat endocrine and reproductive dysfunctions in the PCOS-IR rat model, as well as to compare their effects in improving the clinical and biochemical aspects of PCOS with those of metformin alone and in combination with SeNPs.

It is worth noting that IR has been linked to PCOS prevalence and progression. As a result, PCOS-IR has been a focus of endocrine and metabolic research [28]. Letrozole-induced PCOS is a good model, but it has some limitations, such as the lack of metabolic changes. However, several studies have shown that using letrozole in combination with a high-fat diet was able to establish a PCOS-IR rat model that showed signs of polycystic ovarian alterations as well as endocrine and metabolic disturbances [29].

In the present study, SeNPs were chosen as a potential therapy for PCOS. Selenium (Se) is an essential trace element with critical biological functions for human health. Se has been revealed to play a crucial role in the function of the islets of Langerhans, the gastrointestinal system, and the ovaries [30]. The current study on the nanosize of Se was driven by the expanding awareness of nanotechnology and the reported biological potentials of nano-sized materials in alleviating a variety of diseases. SeNPs have sparked a lot of interest in treating metabolic disorders because of their unique biological functions [31].

Undoubtedly, weight control is beneficial to ovulation recovery and IR remission in PCOS patients; thus, weight reduction is a significant target of PCOS clinical management [32]. Our data revealed that all rats showed the highest weight gain throughout the period of HFD compared to those receiving the control diet. Following that, untreated PCOS-IR rats showed significant weight gain and increased Lee’s index, providing the first indicator of metabolic derangements in the PCOS-IR model group. HFD resulted in a significant increase in fat mass. On the other hand, treatment with SeNPs and/or MET significantly reduced weight gain and Lee’s index of rats. Their influence on body weight may be due to anorexigenic impact as well as their insulin sensitizing effect [33,34].

Interestingly, IR is thought to be the pathological cornerstone of PCOS and has been proven to be the primary cause of abortion in PCOS patients. Therefore, improving the degree of IR in PCOS will enhance reproductive capacity and lower the risk of long-term PCOS complications [29]. Interestingly, glucose metabolism is impaired in PCOS due to IR and pancreatic β- cell dysfunction [35]. Furthermore, high androgen levels in PCOS cause IR by perpetuating a chronic inflammatory state [36].

Our data revealed that untreated PCOS-IR rats recorded a significantly high fasting blood glucose level with a concomitant marked increase in fasting insulin level and HOMA-IR. In IR, insulin capacity for enhancing glucose uptake and utilization is diminished. To preserve glycemic control, insulin is oversecreted as a compensatory mechanism, eventually resulting in hyperinsulinemia [37].

On the other hand, SeNPs and/or MET treatment improved glycemic control. Se can elicit insulin-like effects by activating Akt and other kinases in the insulin signalling cascade [38]. Other mechanisms could support the hypoglycemic action of Se, such as inhibition of intestinal glucose transport and acceleration of renal glucose excretion in rats [39]. Moreover, glutathione peroxidase (GPx) expression has also been linked to its hypoglycemic action. GPx protects intranuclear musculoaponeurotic fibrosarcoma oncogene homolog A (MafA), allowing it to transactivate the insulin gene by reducing oxidative stress in the β-cell. Intranuclear MafA has been identified as a transcription factor important in the regulation of the insulin gene [40].

Moreover, the administration of SeNPs and/or MET attenuated IR in experimental animals, confirming the fact that SeNPs and MET have a synergistic therapeutic effect against IR and the ability to improve β-cell functions. These findings matched a recent study that showed that the combination of SeNPs and MET alleviated the majority of diabetic complications and IR in a high-fat diet/streptozotocin-induced type 2 diabetic rat model [41].

From another aspect, dyslipidemia is frequent in PCOS women. Most PCOS patients have impaired lipid profile primarily due to increased testosterone levels, which have been linked to reduced HDL-C and a diminished catabolic removal of LDL-C via binding with androgen receptors [42]. Our results reported a marked increase in serum TG, TC, and LDL-C levels and a decrease in HDL-C levels in untreated PCOS-IR rats. However, administration of SeNPs significantly improved lipid profile, with a significant increase in HDL-c levels compared to the untreated PCOS-IR rats. The reduction in TG and TC levels may be due to the action of HDL-c, which may increase the efflux of TG and TC to hepatocytes for degradation [43,44].

Concerning serum reproductive hormonal levels, our results revealed high testosterone and LH hormone levels in the PCOS-IR group. The androgen level was increased due to inhibiting the activity of cytochrome P450 aromatase, which is required for the aromatization of testosterone to estradiol. At the same time, testosterone and LH levels in SeNPs and/or MET-treated groups are lower than in the PCOS-IR group. Interestingly, estradiol and FSH levels increased significantly in SeNPs-treated groups, as Se is crucial for the high release of estradiol by acting on granulosa cells. In line with these results, Basini et al. suggested that Se modulates granulosa cells proliferation and estradiol synthesis in swine granulosa cells, and this effect may be partly mediated by nitric oxide inhibition [45].

Low-grade inflammation has been identified as an important contributing factor in PCOS [46]. It has been reported that TNF-α plays a vital role in regulating the production of ovarian steroids, follicular growth, and ovulation [47]. Moreover, high TNF-α level has recently been linked to the development of IR and hyperandrogenism [48]. In the current study, TNF-α level was significantly increased in the PCOS-IR group but effectively reduced in SeNPs and/or MET-treated groups proving the significant anti-inflammatory effect of SeNPs that has been documented in previous studies [49].

Oxidative stress has been identified as a major contributor to PCOS which is linked to IR and impaired steroidogenesis by increasing reactive oxygen species and activating cytokine production, causing irregular menstrual cycle and metabolic activity [48]. In our study, the PCOS-IR rats demonstrated a significant increase in ovarian oxidative stress markers (MDA level) and a decrease in anti-oxidant defence indicators (GSH level and SOD activity) in accordance with previous research [50]. Following treatment with SeNPs and MET, these abnormalities were adjusted, restoring the anti-oxidant activity and attenuating the increased oxidative stress markers. Numerous studies have demonstrated that SeNPs have a potent anti-oxidant activity [51,52].

Mitochondria could be the primary organelles responsible for disturbed energy metabolism in obese PCOS patients. Obesity-induced lipid accumulation in the ovaries disrupts oocyte metabolism and impairs mitochondrial function. Compared to oocytes from mice fed a normal diet, oocytes from obese mice fed a high-fat diet have abnormal morphologies, lower levels of ATP and higher levels of ROS [53].

Moreover, IR and hyperandrogenism are inextricably linked to mitochondrial dysfunction. Insulin is the primary regulator of oxidative phosphorylation, and its secretion may have an immediate impact on mitochondrial function. On the other side, mitochondria are critical to insulin's normal function. Increased ROS can cause abnormal activation of serine/threonine kinase signalling pathways and increase phosphorylation of insulin receptors, resulting in decreased insulin effectiveness [54]. As a result, mitochondrial dysfunction may be the root cause of IR and PCOS complications. Previous studies have revealed that mitochondrial dysfunction, along with an impaired anti-oxidant system, may indeed be involved in the pathophysiology and progression of PCOS-IR [55].

To determine if the administration of SeNPs and/or MET improved mitochondrial function, we assessed MMP, ATP level, and complex-I activity in control, PCOS-IR, and treatment groups. In the PCOS-IR group, mitochondrial function was impaired, as evidenced by a decrease in MMP, ATP level, and impairment of mitochondrial complex I activity as the increased ROS production impairs mitochondrial function. Our data revealed that treated groups demonstrated mitochondrial function recovery, including significant improvement of MMP, ATP level and complex I activity with marked correction in the combined treatment group. In line with these results, SeNPs increased ATP level and enhanced the activity of mitochondrial complexes in the kidney in experimental rats with vancomycin-induced nephrotoxicity [56].

Also, traditional Chinese medicine active ingredients-based SeNPs exhibited protective effects against oxidative stress-induced cytotoxicity in PC12 cells through alleviating mitochondrial dysfunction [57]. Moreover, MET treatment improved mitochondrial function in leukocytes of PCOS patients [58].

The PI3K/Akt signalling pathway is part of the post-insulin receptor signal pathway that has a crucial role in follicular growth and development. Dysfunctional PI3K/Akt pathway has been associated with impaired glucose utilization, IR in the ovaries, and anovulation in PCOS [59]. Our data showed that PI3K and Akt expressions were significantly downregulated in the whole ovarian tissue of the PCO-IR group compared to control rats. Whereas they were upregulated in the ovarian tissue of SeNPs and/or MET treatment groups. According to Qiu et al., Liuwei Dihuang pills improved IR by acting on the PI3K/Akt signalling pathway, thus ameliorating PCOS symptoms [7].

Similarly, Zhang et al. proved that induction of the PI3K/Akt pathway is essential for promoting ovarian reserve and fertility in mice with premature ovarian failure [60]. These findings suggested that the PI3K/Akt signalling pathway may be implicated in how SeNPs regulate ovarian functions in PCO-IR rats. However, the specific mechanism requires further investigation.

Interestingly, Ki-67 is an antigen present in proliferating cells at various cell cycle stages and is absent in cells that are arrested in the G0 phase [61]. Our results showed a significant increase in Ki-67 immuno-expression was detected in the granulosa cells of the groups treated with SeNPs and/or MET, reflecting the increased number of granulosa cells after treatment. In line with these findings, a previous study discovered that treatment with SeNPs increased Ki-67 expression and, thus, increased cellular proliferation and spermatogenesis in gentamicin-induced toxicity in rat testis [62]. Besides, Lombardi et al. reported increased Ki-67 expression in the granulosa cell layer in a PCOS rat model [63].

**Limitation:** A potential limitation of our study was that we have estimated the RNA expression in the ovarian tissue as a whole, and the difference in gene expression between different groups was attributed to the treatment. Further studies are still needed to evaluate whether the changes in gene expression are linked to the decrease in cysts density or causally related to this process due to the intervention to determine the precise mechanism.

## 5. Conclusion

In conclusion, this study found that the PI3K/Akt pathway plays an important role in the metabolic disturbances associated with PCOS. It also demonstrated that SeNPs have a considerable promising effect of enhancing PCOS by regulating PI3K/Akt pathway, thereby improving insulin sensitivity which is a key factor in PCOS complications. It also helped with reproductive hormonal imbalance and dyslipidemia, which are frequent in PCOS women. Besides, SeNPs displayed potent anti-oxidant activity in the ovaries, then restored mitochondrial function and contributed significant anti-inflammatory activity by controlling inflammatory cytokines. Furthermore, SeNPs combined with MET had a greater effect on the values of these parameters than either SeNPs or MET alone, proving that SeNPs and MET may have a synergistic therapeutic effect on patients with PCOS-IR. Therefore, more research into the underlying mechanisms, safety, and potential therapeutic use of SeNPs against the pathophysiology of PCOS is strongly encouraged.

## Availability of data and material

The authors declare that data supporting the findings of this study are available within the article.
